# Investigating the Epigenetic Effects of a Prototype Smoke-Derived Carcinogen in Human Cells

**DOI:** 10.1371/journal.pone.0010594

**Published:** 2010-05-12

**Authors:** Stella Tommasi, Sang-in Kim, Xueyan Zhong, Xiwei Wu, Gerd P. Pfeifer, Ahmad Besaratinia

**Affiliations:** 1 Department of Cancer Biology, Beckman Research Institute of the City of Hope National Medical Center, Duarte, California, United States of America; 2 Division of Information Sciences, Beckman Research Institute of the City of Hope National Medical Center, Duarte, California, United States of America; Health Canada, Canada

## Abstract

Global loss of DNA methylation and locus/gene-specific gain of DNA methylation are two distinct hallmarks of carcinogenesis. Aberrant DNA methylation is implicated in smoking-related lung cancer. In this study, we have comprehensively investigated the modulation of DNA methylation consequent to chronic exposure to a prototype smoke-derived carcinogen, benzo[*a*]pyrene diol epoxide (B[*a*]PDE), in genomic regions of significance in lung cancer, in normal human cells. We have used a pulldown assay for enrichment of the CpG methylated fraction of cellular DNA combined with microarray platforms, followed by extensive validation through conventional bisulfite-based analysis. Here, we demonstrate strikingly similar patterns of DNA methylation in non-transformed B[*a*]PDE-treated cells *vs* control using high-throughput microarray-based DNA methylation profiling confirmed by conventional bisulfite-based DNA methylation analysis. The absence of aberrant DNA methylation in our model system within a timeframe that precedes cellular transformation suggests that following carcinogen exposure, other as yet unknown factors (secondary to carcinogen treatment) may help initiate global loss of DNA methylation and region-specific gain of DNA methylation, which can, in turn, contribute to lung cancer development. Unveiling the initiating events that cause aberrant DNA methylation in lung cancer has tremendous public health relevance, as it can help define future strategies for early detection and prevention of this highly lethal disease.

## Introduction

Lung cancer is the chief cause of cancer-related mortalities, worldwide [1], [2]. The death toll of lung cancer is estimated to reach 1.5 millions in 2010 [2]. The projection of the enormous global burden of this malignancy in the 21^st^ century underscores the significance of this disease as an ominous public health problem. Etiologically, tobacco smoking continues to represent the single most important risk factor for lung cancer development [2]. Although the initial flurry of research has unraveled many aspects of smoke-derived lung carcinogenesis, the exact underlying mechanism of this malignancy awaits further delineation [3], [4]. The gaps in mechanistic knowledge of smoke-associated lung cancer constitute the main obstacle in the management of this disease, which is currently diagnosed mostly at late stages with poor response to surgery, chemotherapy, and/or radiotherapy that leads to high mortality [3]. Elucidation of the underlying mechanism of smoke-induced lung carcinogenesis can help define future strategies for early diagnosis, prognosis, treatment, and prevention of lung cancer [4].

Epigenetic mechanisms of carcinogenesis manifest as heritable changes in gene expression without involving alterations in the underlying DNA sequence [5], [6], [7]. Aberrant DNA methylation is the best-studied epigenetic mechanism, and causally implicated in human cancer [5], [6]. A global loss of DNA methylation (*hypo*methylation) and a locus/gene-specific gain of DNA methylation (*hyper*methylation) are two distinct hallmarks of carcinogenesis [7], [8]. Whereas DNA hypomethylation is thought to contribute to oncogenesis by reactivation of latent retrotransposons, induction of genomic instability, and activation of protooncogenes [9], [10], DNA hypermethylation is believed to elicit tumorigenesis by transcriptional silencing of tumor suppressor genes [5], [6], [7], [8]. Aberrant DNA methylation occurs predominantly in the context of 5′-CpG-3′ dinucleotides (CpGs) [5], [6], [7], [8]. In mammalian genomes, the vast majority of CpGs are normally methylated, *e.g.*, 80–90% of CpGs in the human genome are methylated [5], [6], [7]. The remaining methylation-free CpGs are found in stretches of >500 base pairs (bp) with a GC content of >55% and an observed/expected CpG ratio of ≥0.65, conventionally termed “CpG islands” [11]. Of significance is the genomic locations of CpG islands, which often span the 5′ end region (promoter, untranslated region and exon 1) of many genes, *e.g.*, ∼70% of all human promoters encompass CpG islands [5], [6], [7]. Hypermethylation of CpG islands in the promoter regions of tumor suppressor genes concomitant with their transcriptional silencing have been observed in virtually all types of human cancer, including various smoking-related malignancies [5], [6], [7], [8], [12], [13]. Global DNA hypomethylation has also been found in a variety of human cancers, albeit with ambiguous link to smoking *per se* [14], [15], [16], [17].

Polycyclic aromatic hydrocarbons (PAH) are a prominent class of carcinogenic compounds present in tobacco smoke, as well as in numerous other sources, including occupational, environmental, *e.g.*, dietary, and medicinal sources [18]. Benzo[*a*]pyrene (B[*a*]P) is a prototype PAH, which requires metabolic activation to its ultimate carcinogenic form, B[*a*]P diol epoxide (B[*a*]PDE), to exert its biological effects *in vivo* [18]. In the early 1980 s, a few epigenetic studies have used B[*a*]P and/or B[*a*]PDE, as model tobacco-smoke carcinogens, to investigate the modulation of DNA methylation *in vitro* [19], [20], [21]. Modification of DNA with B[*a*]PDE resulted in impairment of DNA methyltransferase (DNMT) activities, manifested as inhibition of catalyzing reaction between the methyl donor *S*-adenosylmethionine (SAM) and the substrate DNA [19], [20], [21]. Furthermore, treatment of murine cell lines C3H/10T1/2 and BALB/3T3 A31 with B[*a*]P caused a reduction in the 5-methylcytosine content of cellular DNA, albeit only in the latter cell line [19]. More recently, two other studies have investigated the effects of these chemicals on DNA methylation in MCF-7 breast cancer cells [22] and immortalized bronchial epithelial cells [23]. Applying a restriction enzyme-based, polymerase chain reaction (PCR) –dependent microarray approach, non-conclusive and counterintuitive results were obtained regarding the methylation status of a subset of human CpG islands interrogated in the former study [22]. Despite no alteration in mRNA expressions of the maintenance *DNMT1* or the *de novo DNMT3a* or *DNMT3b*, there were increased levels of DNMT1 protein and promoter hypermethylation of several genes of the panel of 30 genes analyzed in the latter study [23]. Altogether, it would be oversimplistic, however, to expect a direct link between DNA methylation status and *DNMTs*, either at the expression or activity level [24], [25], [26], [27], considering the simultaneous occurrence of global DNA hypomethylation and region-specific DNA hypermethylation in cancer [5], [6], [7], [24], [25]. The modulation of DNA methylation consequent to carcinogen exposure, therefore, should be investigated by cataloguing DNA methylation profile, on a genome-wide scale or in genomic regions of potential significance in cancer, preferably in ‘normal’ human cells challenged with carcinogens. To date, the current literature lacks a comprehensive study of such design, however.

We have recently developed a versatile DNA methylation detection method, the methylated-CpG island recovery assay (MIRA), in combination with microarray platforms [28], which enables analysis of DNA methylation status in individual genes as well as in large number of genes, genome-wide [12], [13], [29], [30]. As a pulldown assay for enrichment of the methylated CpG content of cellular DNA, the MIRA is based on the ability of the methyl-CpG binding (MBD) proteins, the MBD2b/MBD3L1 complex, to specifically bind methylated-CpG dinucleotides [28], [31]. The MIRA-enriched DNA fraction, without undergoing restriction enzyme digestion or PCR amplification, can be fluorescently labeled and hybridized to commercially available CpG island/genome tiling arrays [28]. In the present study, we have used a MIRA-assisted microarray approach to establish DNA methylation profiles in normal human fibroblasts chronically exposed to B[*a*]PDE *in vitro*. For verification purposes, we have scrutinized the data obtained by our MIRA-assisted microarray analysis using the conventional combined bisulfite-restriction analysis (COBRA) [32], and the gold standard of DNA methylation analysis, sodium bisulfite genomic sequencing [33]. Here, we have specifically scanned chromosomal gene-rich regions of very frequent allele loss in lung tumors [34], as well as long- and short interspersed nuclear elements (LINE and SINE, respectively), and long terminal repeat (LTR) retrotransposons, and segmental duplications whose activation through hypomethylation relates to genomic instability and lung cancer [13], [35], [36].

## Results

### Efficiency of carcinogen treatment

Using a well-defined validated cell culture model system and under strictly controlled experimental conditions, we have investigated the modulation of DNA methylation consequent to chronic exposure to the smoke-derived activated carcinogen, B[*a*]PDE. To fairly mimic a real life situation, we treated the cells repeatedly with biologically effective doses of B[*a*]PDE on a daily basis with 3-day-intervals in between the treatments. Of significance, we ensured that the administered doses of B[*a*]PDE did not severely affect the proliferative capacity of the cells because the maintenance of DNA methylation pattern is dependent upon DNA replication during cell division [5], [6], [7], [8]. As shown in Figure S2, we verified the efficiency of carcinogen treatment in our model system by confirming the interaction of B[*a*]PDE with cellular DNA in carcinogen-treated normal human fibroblasts. In all cases, proliferatively-competent cell cultures treated with B[*a*]PDE did reach nearly full confluency, and required multiple rounds of passaging during the course of treatment.

### High-throughput DNA methylation cataloging

**Figure 1:**
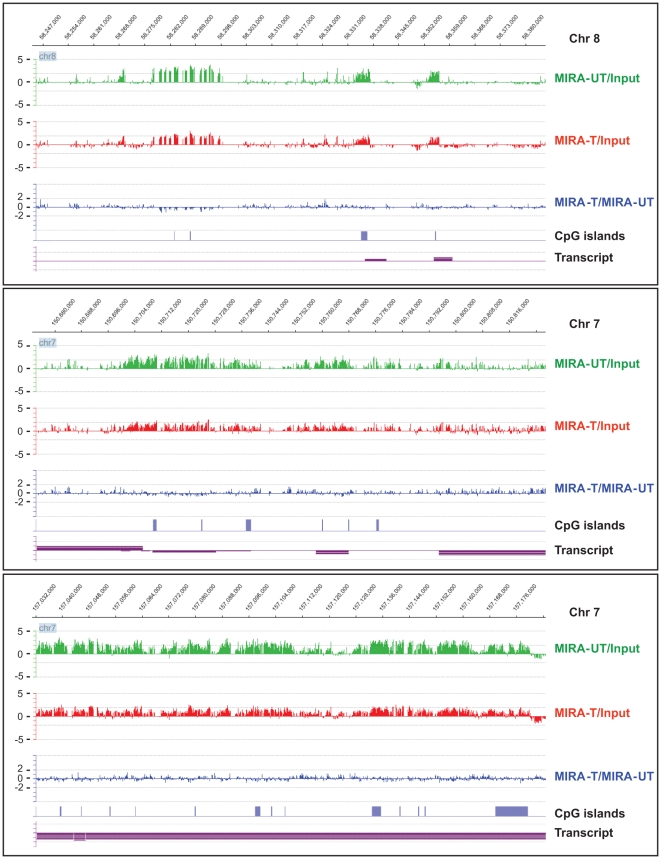
Comparison of DNA methylation profiles between B[*a*]PDE-treated cells and control by MIRA-assisted microarray analysis. Genomic DNA of normal human fibroblasts chronically treated with B[*a*]PDE *vs* DMSO was subjected to MIRA-assisted microarray analysis, as described in the text. Representative methylation array profiles from different chromosomal regions are displayed with corresponding genomic coordinates (indicated on the top). MIRA-T/MIRA-UT'  =  MIRA-enriched B[*a*]PDE-treated DNA *vs* MIRA-enriched DMSO-treated DNA, ‘MIRA-T/Input’  =  MIRA-enriched B[*a*]PDE-treated DNA *vs* Input non-enriched B[*a*]PDE-treated DNA, and ‘MIRA-UT/Input’  =  MIRA-enriched DMSO-treated DNA *vs* Input non-enriched DMSO-treated DNA.

Using NimbelGen tiling array (Roche NimbleGen, Inc., Madison, WI), we have established the status of DNA methylation in chromosomes 7 and 8 in B[*a*]PDE-treated normal human fibroblasts, applying the MIRA-assisted microarray approach. As illustrated in Figure S1, we utilized three different hybridization designs, including (I) MIRA-enriched B[*a*]PDE-treated DNA *vs* MIRA-enriched DMSO-treated DNA, (II) MIRA-enriched B[*a*]PDE-treated DNA *vs* Input non-enriched B[*a*]PDE-treated DNA, and (III) MIRA-enriched DMSO-treated DNA *vs* Input non-enriched DMSO-treated DNA. No PCR amplification was performed on the MIRA-enriched fractions before hybridization to the arrays. Applying very stringent bioinformatics criteria, we made comparative analysis between DNA methylation patterns found in various genomic regions in B[*a*]PDE-treated cells *vs* control. Overall, we observed strikingly similar patterns of DNA methylation in B[*a*]PDE-treated cells *vs* control. The remarkable resemblance of DNA methylation status between B[*a*]PDE-treated cells and control is shown at different representative genomic regions in Figure 1 and Figure S3. Marginal differences in DNA methylation patterns found at certain loci in B[*a*]PDE-treated cells *vs* control were deemed non-significant after statistical analysis. On average, the most pronounced fold-difference in the extent of DNA methylation between B[*a*]PDE-treated cells and control, as indicated by peaks, for example in Figure 1 and Figure S3, did not exceed 1.66 for hypermethylated targets. No hypomethylated targets, even at a fold-difference level of 1.50, was detectable in B[*a*]PDE-treated cells *vs* control. For comparison, we have previously established the profile of DNA methylation in smokers' lung tumors *vs* adjacent non-tumorous tissues, as determined by parallel analysis [13]. In the latter case, the fold-differences (tumor *vs* normal lung) in the extent of DNA methylation reached more than 10 for several hundred hypermethylated targets, and more than 3 for several thousand hypomethylated targets [13]. Of note, we have also repeated the above analysis using the promoter CpG island microarrays (Agilent Technologies Inc.), which cover virtually the entire set of CpG islands of the human genome. Similarly to results obtained by the chromosomal tiling arrays, we did not find any significant difference in the extent of CpG islands methylation between B[*a*]PDE-treated cells and control (*data not shown*).

Furthermore, we performed an electromobility shift assay [28] to determine the affinity of the MBD2b/MBD3L1 complex for methylated CpGs in the presence and absence of B[*a*]PDE-DNA adducts. The latter was to rule out the possibility that B[*a*]PDE-DNA adduction at methylated CpGs may adversely affect the formation of MBD2b/MBD3L1 complex at these dinucleotides, thus, impeding the MIRA pulldown procedure. As shown in Figure S4, we found invariable formation of the MBD2b/MBD3L1 complex in a 55-mer methylated CpG containing-oligonucleotide, in the presence and absence of B[*a*]PDE-DNA adducts.

### Conventional DNA methylation profiling

**Figure 2:**
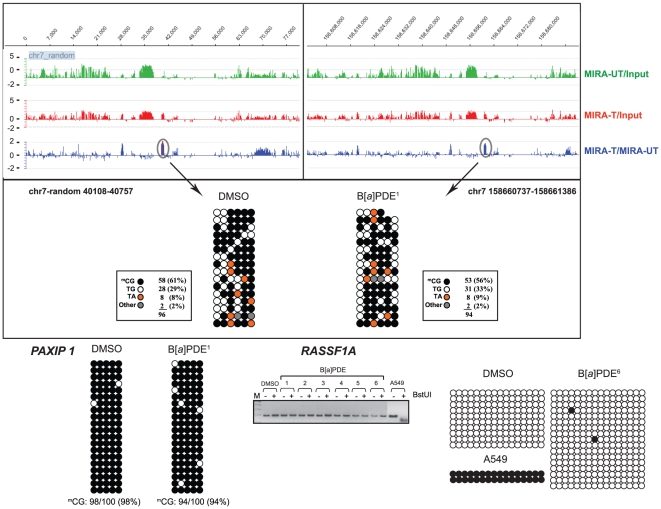
Locus/gene-specific verification of DNA methylation profiles in B[*a*]PDE-treated cells *vs* control by COBRA and bisulfite genomic sequencing. Differentially, yet marginally, methylated target loci/genes identified by MIRA-assisted microarray analysis in B[*a*]PDE-treated cells *vs* control, were selected randomly, and subjected to conventional COBRA [32] and bisulfite sequencing analyses [33] to establish their methylation status, individually. The lack of ‘hypermethylation’ in the specified targets was confirmed by the COBRA [32] and/or genomic sequencing [33] methods. For comparison, we have presented readily detectable hypermethylation of one of these targets (*RASSF1A*) in A549 lung cancer cell line. Data from independent B[*a*]PDE-treated samples, indicated by superscript numbers, *e.g.*, B[*a*]PDE^1^, are shown. UT  =  DMSO-treated DNA; T  =  B[*a*]PDE-treated DNA. (•)  =  Methylated CpG; (○)  =  Unmethylated CpG; ^m^CG: Absolute number of methylated CpGs/total CpGs (% methylated CpGs); None of the differences in ^m^CG% between B[*a*]PDE-treated DNA *vs* control was statistically significant (Fisher's exact test).

We validated the data obtained by MIRA-assisted microarray analysis using the conventional COBRA assay [32] and bisulfite genomic sequencing [33]. We randomly selected differentially, yet marginally, methylated target loci/genes identified by the above analysis in B[*a*]PDE-treated cells *vs* control, and established their methylation status, individually. In agreement with our MIRA-assisted microarray data, both the COBRA [32] and bisulfite genomic sequencing [33] analyses showed no significant difference in the profile of DNA methylation between B[*a*]PDE-treated cells and control for all the analyzed targets. As shown in Figures 2–5, there were remarkably similar patterns of DNA methylation in all the examined targets in B[*a*]PDE-treated cells *vs* control.

**Figure 3 pone-0010594-g003:**
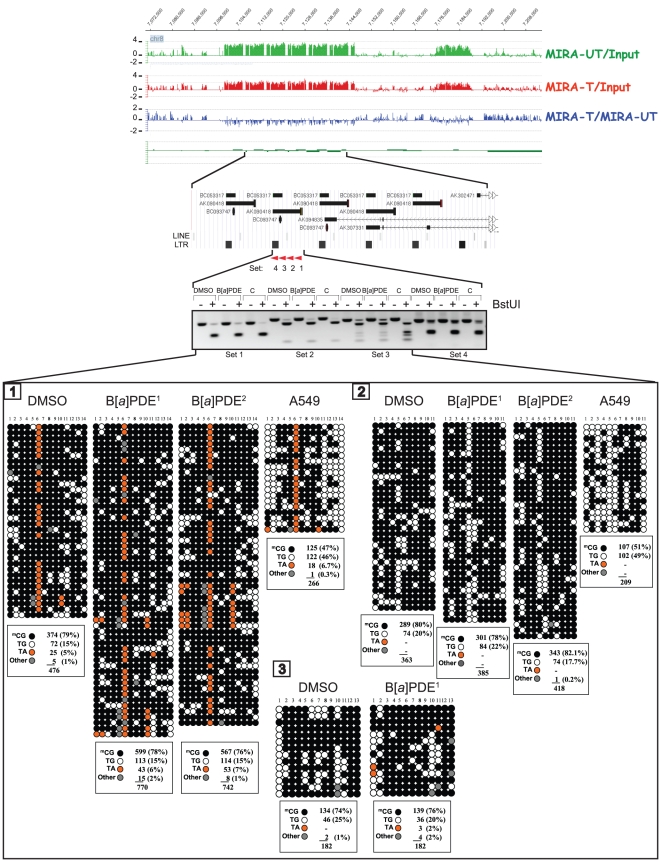
Locus/gene-specific verification of DNA methylation profiles in B[*a*]PDE-treated cells *vs* control by COBRA and bisulfite genomic sequencing. The lack of ‘hypomethylation’ in the segmental duplications encompassing LINE and LTR retrotransposons was confirmed by the COBRA [32] and genomic sequencing [33] methods. For comparison, we have presented readily detectable hypomethylation of these repetitive DNA elements in A549 lung cancer cell line. None of the differences in ^m^CG% between B[*a*]PDE-treated DNA *vs* control was statistically significant (Fisher's exact test). C  =  HeLa DNA methylated *in vitro* with M. SssI CpG methyltransferase. (*See*, also legend for Fig. 3).

**Figure 4 pone-0010594-g004:**
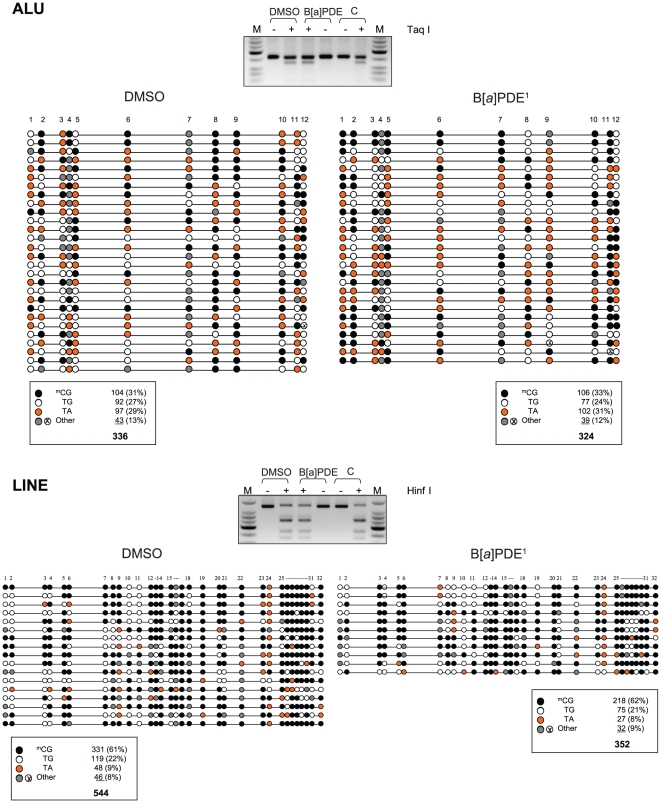
Locus/gene-specific verification of DNA methylation profiles in B[*a*]PDE-treated cells *vs* control by COBRA and bisulfite genomic sequencing. The lack of ‘hypomethylation’ in the SINE (ALU) and LINE retrotransposons was confirmed by the COBRA [32] and genomic sequencing [33] methods; none of the differences in ^m^CG% between B[*a*]PDE-treated DNA *vs* control was statistically significant (Fisher's exact test) (*See*, also legends for Fig. 3 and 4). M =  Size marker.

**Figure 5:**
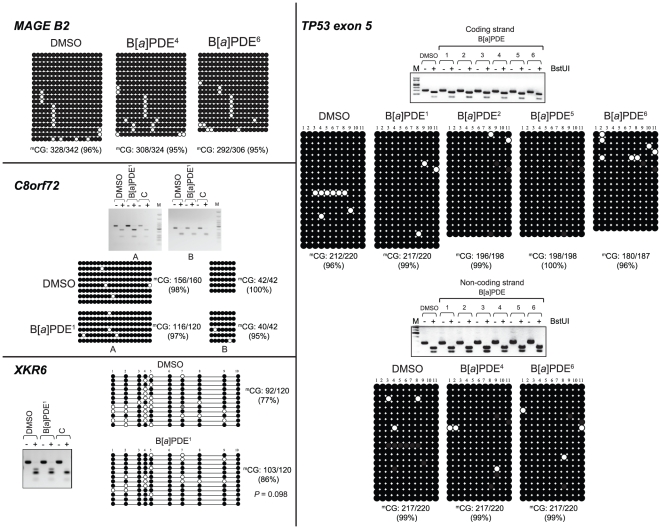
Locus/gene-specific verification of DNA methylation profiles in B[*a*]PDE-treated cells *vs* control by COBRA and bisulfite genomic sequencing. The lack of ‘hypomethylation’ in the specified targets was confirmed by the COBRA [32] and genomic sequencing [33] methods; none of the differences in ^m^CG% between B[*a*]PDE-treated DNA *vs* control was statistically significant (Fisher's exact test) (*See*, also legends for Figs. 3–5).

To specifically determine DNA hypomethylation events consequent to carcinogen treatment, we investigated the methylation status of LINE, SINE, and LTR retrotransposons, and segmental duplications in bisulfite-treated DNA from B[*a*]PDE-treated cells *vs* control. We adapted a published procedure [37], which involves primer amplification of the consensus sequences from the respective elements followed by appropriate restriction enzyme digestion or direct sequencing. Evolutionarily, methylated CpGs on the forward- or reverse strands of these elements can undergo spontaneous deamination of 5-methylcytosine to thymine, thereby mutating to 5′-TpG-3′ or 5′-CpA-3′, respectively. Dependent on the status of cytosine methylation, non-mutated CpGs can be converted to 5′-TpG-3′ (if unmethylated) or remain unchanged (if methylated) after bisulfite treatment of DNA *in vitro* (*see*, Fig. S5) [37]. Whereas restriction enzyme digestion of bisulfite-treated and PCR-amplified DNA can help differentiate between methylated CpGs and unmethylated and/or mutated CpGs, direct genomic sequencing will provide detailed information on the status of CpG methylation and mutation in these elements [13], [37]. As shown in Figures 3 and 4, neither restriction enzyme digestion- nor direct sequencing of bisulfite-treated and PCR-amplified fragments derived from these elements showed any significant difference in the extent of CpG methylation between B[*a*]PDE-treated cells and control. For comparison, we have presented readily detectable hypomethylation of these elements in A549 lung cancer cell line, as determined by parallel analysis (*see*, Fig. 3).

Because lung cancer is derived from the epithelial compartment of the lung, we also extended our DNA methylation analysis to normal human bronchial epithelial cells (Cambrex, Walkersville, MD) exposed repeatedly to B[*a*]PDE using the same treatment protocol, which was used for normal human fibroblasts (*see*, Material and Methods). As the former cell type was much more sensitive to carcinogen assault, we could maximally treat these cells with 0.2 µM B[*a*]PDE for 6 consecutive daily doses with 3-day-intervals in between the treatments. Similarly to results found in normal human fibroblasts chronically exposed to B[*a*]PDE, we observed no appreciable difference in the extent or profile of DNA methylation between B[*a*]PDE-treated normal human bronchial epithelial cells and control, as determined by our MIRA-based microarray analysis followed by extensive validation through conventional bisulfite-based analysis (*data not shown*).

## Discussion

Aberrant DNA methylation is the most-extensively studied epigenetic mechanism of carcinogenesis [5], [6], [7], [8], and implicitly involved in smoking-related lung cancer [3], [12], [13]. The underlying involvement of aberrant DNA methylation in lung carcinogenesis, in particular in tumor initiation, however, awaits further elucidation [5], [6], [12], [13]. In the present study, for the first time, we have comprehensively investigated the modulation of DNA methylation in normal human cells chronically exposed to a typical smoke-derived carcinogen, B[*a*]PDE [18]. Using our recently developed methylation detection method, the MIRA-assisted microarray approach [28], together with conventional COBRA [32] and bisulfite sequencing [33], we have scanned genomic regions of relevance for lung cancer in normal human cells treated with B[*a*]PDE *in vitro*.

We set up a treatment protocol that resembled - as much as technically possible – a real life situation, in which normal human cells were exposed chronically to biologically effective doses of B[*a*]PDE, while allowing for the potential epigenetic effects to occur in proliferatively-competent cells. Using our high-throughput MIRA-assisted microarray analysis [28], we found remarkably similar patterns of DNA methylation in B[*a*]PDE-treated cells *vs* control. Methodologically, the MIRA enrichment procedure takes advantage of the property of the MBD2b/MBD3L1 complex to specifically bind methylated-CpGs [28], [31]. Of all MBD proteins, MBD2b has the highest affinity for methylated CpGs [38], and the binding reaction is enhanced in the presence of the MBD3L1 protein [28], [31]. As shown in Figure S4, we have empirically ruled out the possibility that B[*a*]PDE-DNA adduction at methylated CpGs may adversely affect the formation of MBD2b/MBD3L1 complex at these dinucleotides. Thus, we verified that MIRA-based analysis is appropriate for studying DNA methylation in B[*a*]PDE-treated cells herein.

Our MIRA-assisted microarray approach is a genome-scale interrogation assay for detecting aberrant DNA methylation, including global hypomethylation and locus/gene specific hypermethylation [12], [13], [28], [29], [31]. Despite being comprehensive, the approach is very straightforward inasmuch as it does not rely on commonly used procedures, such as restriction enzyme digestion or PCR amplification of DNA, for detecting aberrant DNA methylation. The latter two procedures are known to be impeded by the presence of bulky adducts in lesion-bearing DNA [39], [40], [41], [42]. To further provide proof of evidence on the utility of MIRA-assisted microarray approach for characterizing DNA methylation patterns in the genome, we also verified the validity of the data obtained by our MIRA-assisted microarray analysis using the well-established COBRA [32] and bisulfite sequencing methods [33]. As shown in Figures 2–5, we confirmed the validity of MIRA-assisted microarray data by demonstrating that there was no significant difference in DNA methylation profile between B[*a*]PDE-treated cells and control using conventional analysis of the representative targets identified by the high throughput MIRA-based analysis.

Our study is unique in that we have comprehensively investigated the modulation of DNA methylation consequent to exposure to a smoke-derived carcinogen, in genomic regions of significance in lung cancer, in ‘normal’ human cells challenged with relevant doses of carcinogen. Previous studies have implicated a relationship between aberrant DNA methylation and smoking-related lung cancer [14], [15], [16], [17]. However, mechanistic studies have yet to establish the exact nature of this relationship by finding the sequence of events that lead to global loss of DNA methylation and locus/gene-specific gain of DNA methylation, which may, in turn, contribute to lung cancer development. It is conceivable that carcinogen exposure can cause a variety of epigenetic effects, such as histone-modifications and chromatin remodeling, microRNA-derived modulation of gene-expression, etc. [6], [7], [43], [44], which may, secondarily and upon engagement of a parallel transforming event, impact upon DNA methylation. Considering the known genotoxic effects of carcinogens present in tobacco smoke [4], it is also plausible that aberrant DNA methylation associated with lung carcinogenesis [45], [46], [47], may as well be a secondary event that is triggered by, *e.g.*, mutations in crucial genes that can directly or indirectly influence key pathways involved in DNA methylation. It can be envisaged that carcinogen-induced epigenetic or genetic alterations, which can affect the DNA methylation network, *e.g.*, by up- or down-regulating the expression or activities of DNMTs or potential demethylase(s), or alternatively their upstream or downstream regulatory genes, may initiate global DNA hypomethylation and/or region-specific DNA hypermethylation, which can, in turn, give rise to lung tumorigenesis.

Previous studies by others have investigated indirectly and/or non-comprehensively the modulation of DNA methylation consequent to exposure to smoke-related carcinogens [19], [20], [21], [22], [23]. Of concern in these studies are methodological and/or conceptual issues, such as using excessive treatment conditions, *e.g.*, naked DNA treatment with high concentrations of B[*a*]PDE [19], [20], [21], evaluating various proxies for inferring DNA methylation status, *e.g.*, DNMTs activities or expression [19], [20], [21], [23], or assaying cancerous [22] or immortalized cell lines [23] for establishing DNA methylation patterns in a limited number of genes. For example, in studies by Wilson and Jones [19], [20], *in vitro* modification of genomic DNA with extreme doses of B[*a*]PDE resulted in 12 adducts per 10^3^ nucleotides. Such adduct levels of B[*a*]PDE are physiologically not attainable, *e.g.*, leukocytes DNA from average smokers contains ∼3 B[*a*]PDE-DNA adducts per 10^8^ nucleotides [48]. Also, indirect evaluation of DNA methylation status based on proxy quantification cannot provide definitive information as the relationship between such indicators and DNA methylation is less than straightforward [24], [25], [26], [27]. Inherent in model systems that utilize cancerous or immortalized cell lines are the unknowns regarding their “*comparability*” to normal human cells [49]. Additional concerns include technical uncertainties surrounding the applied DNA methylation detection systems. For instance, application of a restriction enzyme-based, PCR–dependent microarray approach for studying DNA methylation in B[*a*]PDE-treated cells has proved unsuccessful [22] due to the potential interference of B[*a*]PDE-DNA adducts with restriction enzyme digestion and/or PCR-amplification steps involved therein [39], [40], [41], [42].

Currently, high throughput next-generation sequencing projects are analyzing large numbers of human lung tumors. These projects are poised to identify unique pathways that are adversely affected in human lung cancer. To infer causality, however, the aberration of these pathways does need to be experimentally recapitulated. For example, it is likely that next-generation sequencing of human lung tumors will elucidate genetic or epigenetic alterations that are specifically associated with exposure to tobacco smoke carcinogens. The relevance of such findings should be verified in validated experimental model systems under well-defined and controlled exposure conditions. As the upcoming data from the sequencing of smokers' lung-cancer genomes and epigenomes will become available, validated model systems should help delineate various aspects of the pathogenesis of this disease. Of importance, genetic or epigenetic mechanisms affecting specific pathways should be investigated so that their role as a driving force behind each individual pathway can be clearly established.

Lastly, we acknowledge that B[*a*]PDE-treatment of normal human cells in the present study is a reasonable recapitulation of chronic exposure to smoke-derived carcinogens, albeit much shorter than what typical smokers' lung cells experience *in vivo*. Here, the resistance of normal human cells to undergo transformation *in vitro* prevented us from examining the possibility that aberrant DNA methylation may occur as a rare stochastic event in individual cells, which might then be selected for through a growth advantage [23]. Admittedly, we should also consider a different scenario, in which smoke-derived carcinogens, other than B[*a*]PDE, are the culprit epimutagens that may cause aberrant DNA methylation in lung carcinogenesis.

In conclusion, we have demonstrated that *in vitro* chronic treatment of normal human cells with a prototype smoke-derived carcinogen, B[*a*]PDE [18], does not result in aberrant DNA methylation in genomic regions of relevance for lung cancer, within a timeframe that precedes cellular transformation. Our data warrant further mechanistic research into the sequence of epigenetic and/or genetic events, which initiate global loss of DNA methylation and locus/gene-specific gain of DNA methylation that may, in turn, contribute to lung cancer development. Identifying the initiating events that cause aberrant DNA methylation in lung cancer has significant public health relevance, as it can help define future strategies for early diagnosis and prevention of this malignancy.

## Materials and Methods

### Ethics Statement

Having read the “*PLoS ONE* Guidelines for Authors”, all the authors of this manuscript confirm that, an ethics statement is not required for this work.

### Cell culture and chemical treatment

The normal human fibroblast cells used in the present study are described in References [50], [51]. Early passage normal human fibroblasts, prepared from neonatal foreskin [50], [51], were grown as monolayer at ∼25% confluence in Dulbecco's Modified Eagle's Medium (DMEM) (Irvine Scientific, Santa Ana, CA) supplemented with 10% fetal bovine serum (FBS). Prior to chemical treatment, the culture media were removed, and the cells were washed thoroughly with phosphate buffered saline (PBS). The culture dishes were filled with serum free DMEM, and subsequently freshly prepared B[*a*]PDE (1 µM) (Midwest Research Institute, Kansas City, MO) or control solvent [dimethylsulfoxide (DMSO)] were added to the media, and incubation was performed at 37°C for 20 minutes in the dark. Immediately after treatment, the cells were washed with PBS, fed with complete growth medium (DMEM plus 10% FBS), and cultivated for 3 days, after which an ensuing round of chemical treatment was carried out, as described above. When reaching approximately 90% confluency, all cultures underwent passaging (1 to 3 split) either 24- or 48 hours post chemical treatment. Three days after the 10^th^ round of B[*a*]PDE treatment, the cells were harvested by trypsinization, and subjected to genomic DNA isolation using the DNeasy purification kit (Qiagen, Valencia, CA). The above-specified treatment protocol was based on our preliminary tests in which we established that normal human fibroblasts well-tolerate multiple rounds of treatment with 1 µM B[*a*]PDE, while having 83–89% survival rate and preserving their proliferation capacity by replicating once every 32–36 hours. All experiments were conducted in triplicate.

### Immuno-dot-blot assay

To verify the efficiency of B[*a*]PDE treatment in normal human fibroblasts, we used a standard immuno-dot-blot assay [52], and confirmed the interaction of this chemical with cellular DNA in carcinogen-treated cells. The immuno-dot-blot assay utilizes the rabbit polyclonal BP1-Ab antibody, which is highly specific for the detection of B[*a*]PDE-DNA adducts [52]. Briefly, heat-denatured genomic DNA from B[*a*]PDE-treated cells *vs* control was dot-blotted onto a nitrocellulose membrane using the Bio-Dot Microfiltration Apparatus (Bio-Rad Laboratories, Life Science Group, Hercules, CA). The membrane was laid over an absorbent paper pre-soaked with 0.4 N NaOH for 20 minutes at room temperature. Subsequently, the membrane was blocked by incubating in phosphate buffered saline plus 0.2% Tween 20 (PBS-T) containing 5% non-fat milk (NFM) at 4°C overnight. After multiple washes with PBS-T, the membrane was incubated with BP1-Ab antibody (diluted 1∶20,000 in PBS-T plus NFM) for 2 hours at room temperature. The membrane was washed thoroughly with PBS-T and further incubated with an anti-rabbit horseradish peroxidase conjugated immunoglobulin (eBioscience, Inc., San Diego, CA) for 1 hour at room temperature (1∶5,000 dilution in PBS-T plus NFM). To reveal peroxidase activity, the membrane was stained with the Enhanced Chemiluminescence Detection System (Amersham Biosciences GE Health Care UK limited, Little Chalfont Buckinghamshire, England) according to the manufacturer's instructions. The stained membrane was exposed to x-ray film, and the relative intensity of luminescence was determined using the Bio-Rad Imaging Equipment applying Quantity One image analyzer (Bio-Rad Laboratories).

### MIRA-assisted microarray analysis

To catalogue DNA methylation profile in chromosomal regions of significance in lung cancer, we performed MIRA-assisted microarray analysis [28] on B[*a*]PDE-treated normal human fibroblasts. We used our recently published protocol with some modifications [30]. Briefly, genomic DNA of B[*a*]PDE-treated cells *vs* control (30 µg each) was fragmented by sonication in a Branson Sonifier (Model 350, Duty Cycle: 40%, Output: 4) for five pulses of five seconds each, and one-minute interval among pulses. The average size of the fragments, determined by electrophoresis on 1.5% agarose gel, was between 500 to 800 bp. Purified GST-tagged MBD2b and His-tagged MBD3L1 proteins (60 µg each) were pre-incubated with a solution containing 10 mM Tris-HCl, pH 7.5, 50 mM NaCl, 1 mM EDTA, 1 mM DTT, 3 mM MgCl_2_, 0.1% Triton-X100, 5% glycerol, 25 mg/ml BSA, and sonicated JM110 ^(*dcm-)*^ bacterial DNA (500 ng) for 20 minutes at 4°C on a rocking platform. The fragmented DNA was then added to the pre-incubated mix, and binding of the MBD2b/MBD3L1 complex to methylated CpGs was achieved after an overnight incubation, as described above. The resultant was mixed with pre-washed MagneGST glutathione particles (Promega, Madison, WI), and purified by magnetic capturing according to the manufacturer's instructions. The enriched MBD2b/MBD3L-bound methylated CpG fraction was further processed using the QIAquick PCR purification kit (Qiagen) to elute the methylated CpG fraction therein.

Subsequently, methylated CpG-enriched DNA fragments (1 µg) from B[*a*]PDE-treated cells *vs* respective DMSO-treated control or input DNA (non-enriched control) were labeled with Cy5-dCTP and Cy3-dCTP (Amersham Biosciences, GE Healthcare UK limited), respectively, using a BioPrime Array CGH Genomic Labeling kit (Invitrogen Corp. Carlsbad, CA) (*see*, Fig. S1 for detailed information on labeling & hybridization scheme). Following a purification step, the samples were mixed and hybridized to NimbleGen tiling arrays (HG18, Set 19, Catalog # C4524-19-01) according to the NimbleGen's ChIP-on-chip protocol (Roche NimbleGen, Inc., Madison, WI). This set of microarrays covers regions of the long arm of chromosome 7, and the entire short arm and part of the long arm of chromosome 8, which contains gene-rich regions of very frequent allele loss in lung tumors [34]. After hybridization, washing and processing, the microarray slides were scanned using an Agilent Scanner (Agilent Technologies Inc., Santa Clara, CA), and images were quantified by NimbleScan v2.5 (Roche NimbleGen, Inc.). A schematic representation of our MIRA-assisted microarray approach is shown in Figure S1. All microarray data are MIAME compliant. The raw microarray data have been deposited in the Gene Expression Omnibus repository, which is a MIAME compliant database, as detailed on the MGED Society website http://www.mged.org/Workgroups/MIAME/miame.html. The accession number for our deposited data is GSE21532.

### Microarray data processing and analysis

#### (I) Identification and annotation of methylated regions

Preprocessing of raw data and statistical analysis were performed as described previously with some modifications [30]. Briefly, Log2 ratios between MIRA-enriched and Input DNA samples were generated using NimbleScan software (Roche NimbleGen, Inc.). Probes were selected as positive if their log2 ratios were above 1 (2-fold enriched). For our analysis, we defined a methylated region of interest (methylation peak) as a region with at least 4 positive probes covering a minimum length of 350 bp allowing one gap. Identified methylation peaks were mapped relative to known transcripts defined in the UCSC genome browser HG18 RefSeq database (http://hgdownload.cse.ucsc.edu/goldenPath/hg18/database/). Methylation peaks falling into 1000 bp relative to transcription start sites were defined as “5′-end peaks”; methylation peaks falling within 1000 bp of RefSeq transcript end sites were defined as “3′-end peaks”, and those falling within gene bodies (from 1000 bp downstream of transcription start to 1000 bp upstream of transcript end) were defined as “intragenic” peaks. Methylation peaks that are not close to any known transcripts were defined as “intergenic.”

#### (II) Identification of hyper- and hypo-methylated regions in B[*a*]PDE treated samples

Hyper- and hypo-methylated regions in B[*a*]PDE treated samples were identified by combining data from all three array designs, including (I) MIRA-enriched B[*a*]PDE-treated DNA *vs* MIRA-enriched DMSO-treated DNA, (II) MIRA-enriched B[*a*]PDE-treated DNA *vs* Input non-enriched B[*a*]PDE-treated DNA, and (III) MIRA-enriched DMSO-treated DNA *vs* Input non-enriched DMSO-treated DNA. First, methylation peaks in B[*a*]PDE treated samples were identified as described above using data on the array comparing MIRA-enriched B[*a*]PDE-treated DNA and Input non-enriched B[*a*]PDE-treated DNA and served as the potential candidates of hyper-methylated regions, which are regions only methylated in B[*a*]PDE-treated samples but not in DMSO-treated samples. The hyper-methylated regions were selected if they satisfied both of the following criteria: 1) the difference between the average log2 ratios of probes within these regions in B[*a*]PDE treated sample (MIRA-enriched B[*a*]PDE-treated DNA *vs* Input non-enriched B[*a*]PDE-treated DNA) and the average log2 ratios of probes in untreated sample (MIRA-enriched DMSO-treated DNA *vs* Input non-enriched DMSO-treated DNA) is more than 1 (2-fold); 2) the average log2 ratios of probes on array comparing MIRA-enriched B[*a*]PDE-treated DNA and MIRA-enriched DMSO-treated DNA were above 1 (2-fold higher comparing MIRA-enriched B[*a*]PDE-treated DNA *vs* MIRA-enriched DMSO-treated DNA). Similar analysis approach was used to identify hypo-methylated regions, except that the methylated regions in untreated sample were used as the starting point to look for difference and signal in MIRA-enriched B[*a*]PDE-treated DNA is more than 2-fold lower than that in MIRA-enriched DMSO-treated DNA.

### COBRA and bisulfite genomic sequencing

To verify the data obtained by MIRA-assisted microarray analysis, we used both the COBRA [32], and bisulfite genomic sequencing techniques [33] to confirm the methylation status of individual target loci/genes identified by the above analysis in B[*a*]PDE-treated human fibroblasts. Briefly, total genomic DNA (1 µg) from B[*a*]PDE-treated cells *vs* control was subjected to sodium bisulfite treatment using the Qiagen EpiTect kit according to the manufacturer's instructions (Qiagen). The purified bisulfite-treated DNA was then analyzed by standard COBRA assay [32]. The primer sequences used for PCR amplification of all analyzed targets are available upon request. HeLa DNA was methylated *in vitro* with M. SssI CpG methyltransferase (New England Biolabs, Ipswich, MA), and served as positive control. For genomic sequencing, the PCR products obtained after bisulfite conversion of genomic DNA were cloned into the TOPO-TA cloning vector (Invitrogen Inc.) according to the manufacturer's instructions. Randomly selected clones from B[*a*]PDE-treated DNA *vs* control were sequenced using an ABI-3730 DNA Sequencer (ABI Prism, PE Applied BioSystems, Foster City, CA).

## Supporting Information

Figure S1 A schematic representation of MIRA-assisted microarray approach. Modification of DNA with B[*a*]PDE is shown by chemical structures bound to the DNA fragments. Methylated and unmethylated CpGs are indicated as black and white lollipops, respectively.
(0.12 MB PDF)

Figure S2 Quantification of B[*a*]PDE-DNA adducts by immuno-dot-blot assay. Normal human fibroblasts were chronically treated in vitro with increasing concentrations of B[*a*]PDE vs control solvent (DMSO). Immediately after the end of last treatment, the cells were harvested and genomic DNA was subjected to immuno-dot-blot assay, as described in the text.
(0.29 MB PDF)

Figure S3 Comparison of DNA methylation profiles between B[*a*]PDE-treated cells and control by MIRA-assisted microarray analysis. Genomic DNA of normal human fibroblasts chronically treated with B[*a*]PDE vs control solvent (DMSO) was subjected to MIRA-assisted microarray analysis, as described in the text. Representative methylation array profiles from different chromosomal regions are shown with corresponding genomic coordinates (indicated on the top). MIRA-T/MIRA-UT' = MIRA-enriched B[*a*]PDE-treated DNA vs MIRA-enriched DMSO-treated DNA, 'MIRA-T/Input' = MIRA-enriched B[*a*]PDE-treated DNA vs Input non-enriched B[*a*]PDE-treated DNA, and 'MIRA-UT/Input' = MIRA-enriched DMSO-treated DNA vs Input non-enriched DMSO-treated DNA.
(0.06 MB PDF)

Figure S4 Affinity of the MBD2b/MBD3L1 complex for methylated CpGs in the presence and absence of B[*a*]PDE-DNA adducts determined by gel mobility shift assay. A 55-mer oligonucleotide, containing 1-10 symmetrically methylated CpG dinucleotides, was treated with increasing concentrations of B[*a*]PDE, and subsequently subjected to electromobility gel shift assay, as described earlier (Rauch et al., 2006). Invariable formation of the MBD2b/MBD3L1 complex in the presence and absence of B[*a*]PDE-DNA adducts is indicated by an arrow. MBD2-Ab = Negative control, co-incubated with polyclonal antibody raised specifically against MBD2b protein. Representative result from the oligonucleotide with 10 methylated CpGs is shown.
(0.23 MB PDF)

Figure S5 Conceptual framework for the methylation detection assay in repetitive DNA elements. The assay is an adaptation of a published procedure (Yang et al., 2004), which involves primer amplification of the consensus sequences from the repetitive DNA elements followed by appropriate restriction digestion or direct sequencing (see, text for detailed information on methodology). Adopted from Ref. (Yang et al., 2004).
(0.03 MB PDF)
