# Supplementary figures and images for: Investigating the Epigenetic Effects of a Prototype Smoke-Derived Carcinogen in Human Cells
Source: PLoS One. 2010 May 12;5(5):e10594. doi: 10.1371/journal.pone.0010594 (PMC2868871; doi:10.1371/journal.pone.0010594)

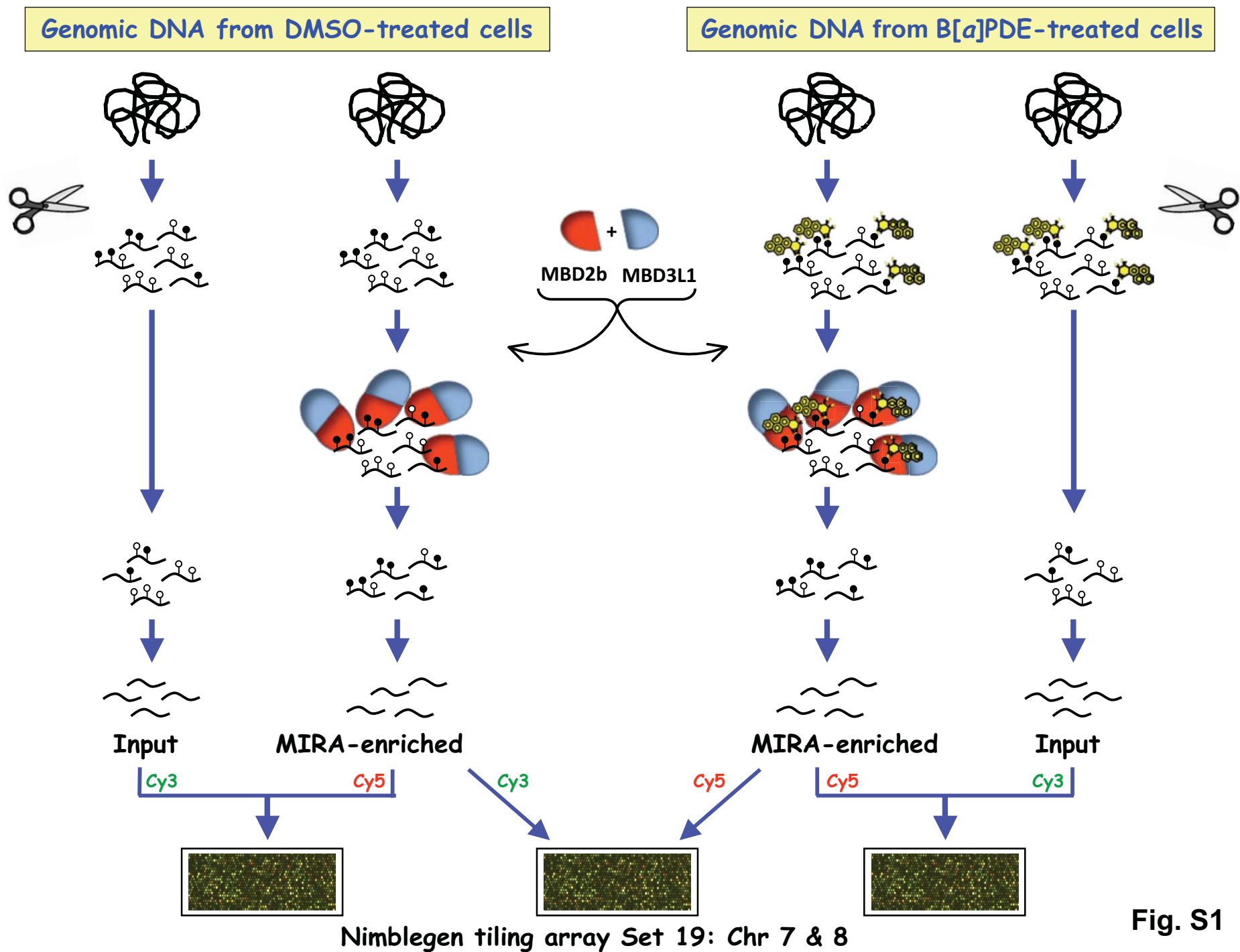

Fig. S1

Supplement: Figure S1 — A schematic representation of MIRA-assisted microarray approach. Modification of DNA with B[a]PDE is shown by chemical structures bound to the DNA fragments. Methylated and unmethylated CpGs are indicated as black and white lollipops, respectively. (0.12 MB PDF) [file pone.0010594.s001.pdf]

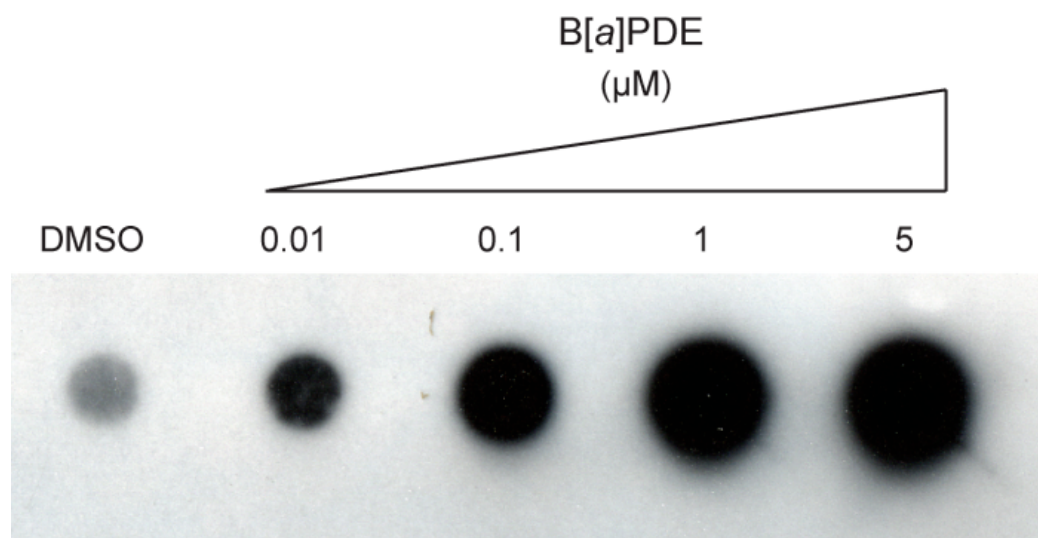

**Fig. S2**

Supplement: Figure S2 — Quantification of B[a]PDE-DNA adducts by immuno-dot-blot assay. Normal human fibroblasts were chronically treated in vitro with increasing concentrations of B[a]PDE vs control solvent (DMSO). Immediately after the end of last treatment, the cells were harvested and genomic DNA was subjected to immuno-dot-blot assay, as described in the text. (0.29 MB PDF) [file pone.0010594.s002.pdf]

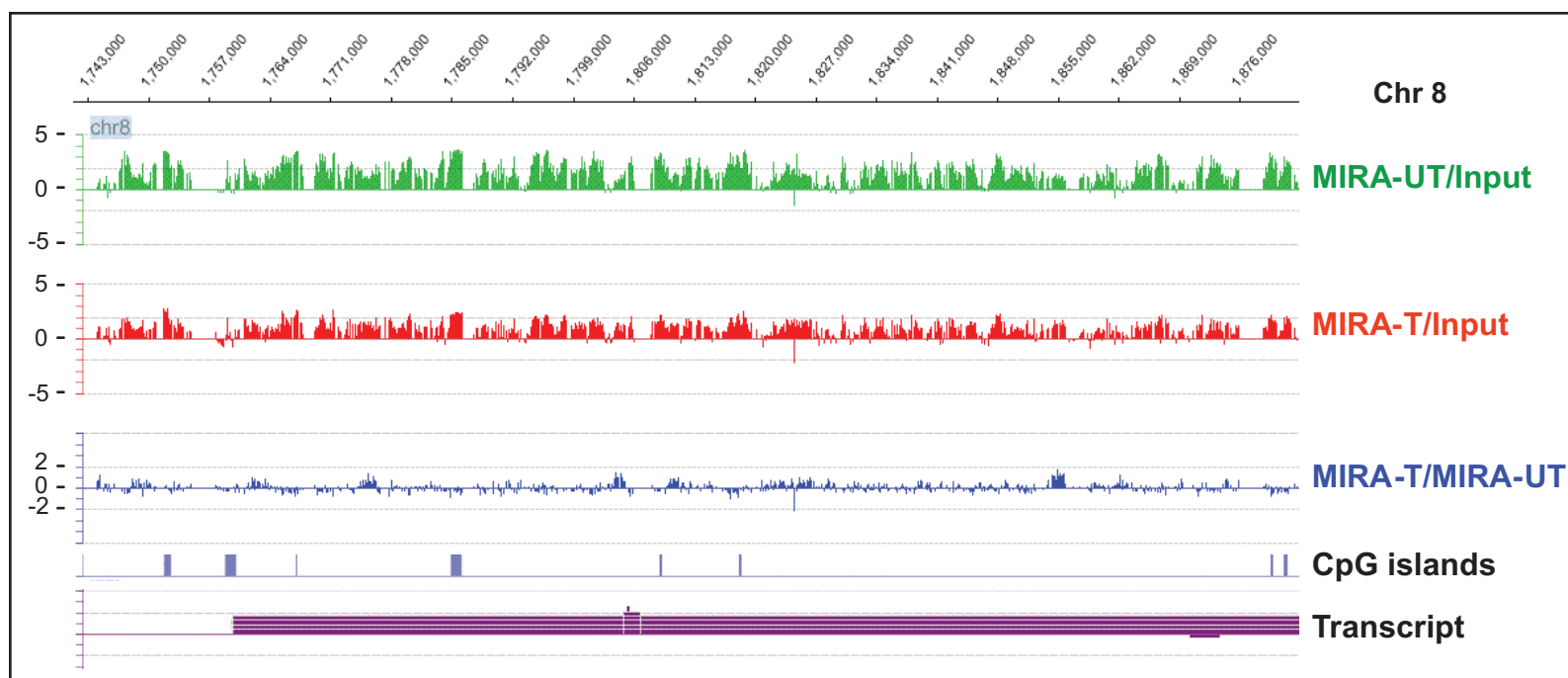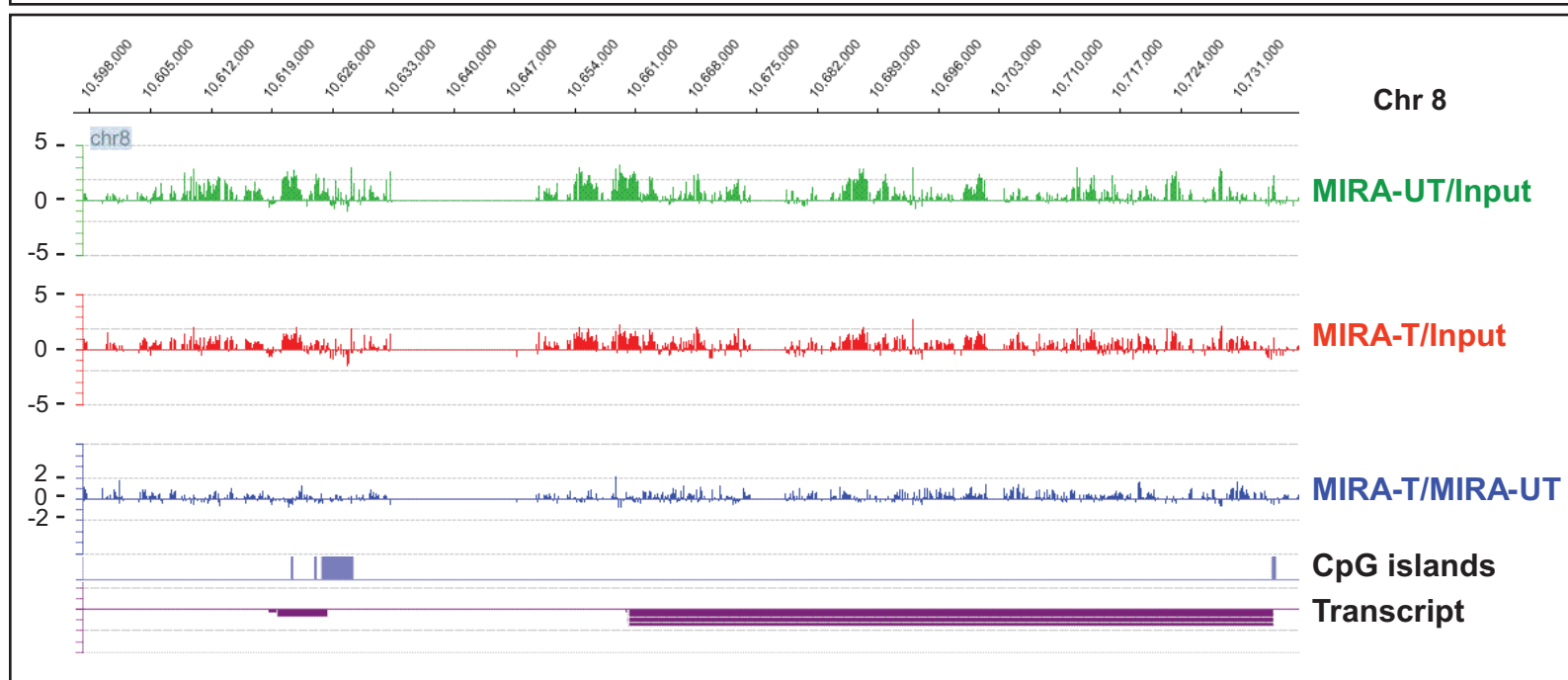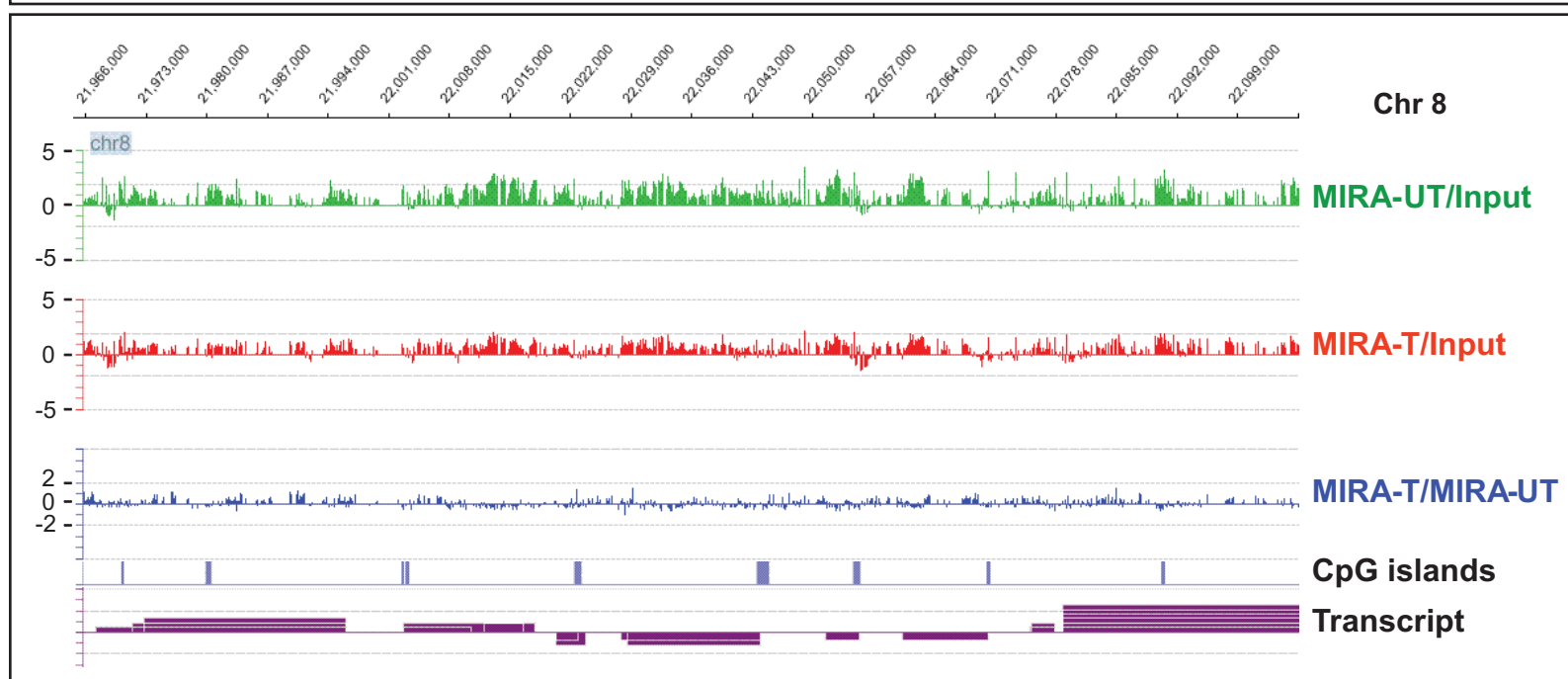

Fig. S3

Supplement: Figure S3 — Comparison of DNA methylation profiles between B[a]PDE-treated cells and control by MIRA-assisted microarray analysis. Genomic DNA of normal human fibroblasts chronically treated with B[a]PDE vs control solvent (DMSO) was subjected to MIRA-assisted microarray analysis, as described in the text. Representative methylation array profiles from different chromosomal regions are shown with corresponding genomic coordinates (indicated on the top). MIRA-T/MIRA-UT' = MIRA-enriched B[a]PDE-treated DNA vs MIRA-enriched DMSO-treated DNA, 'MIRA-T/Input' = MIRA-enriched B[a]PDE-treated DNA vs Input non-enriched B[a]PDE-treated DNA, and 'MIRA-UT/Input' = MIRA-enriched DMSO-treated DNA vs Input non-enriched DMSO-treated DNA. (0.06 MB PDF) [file pone.0010594.s003.pdf]

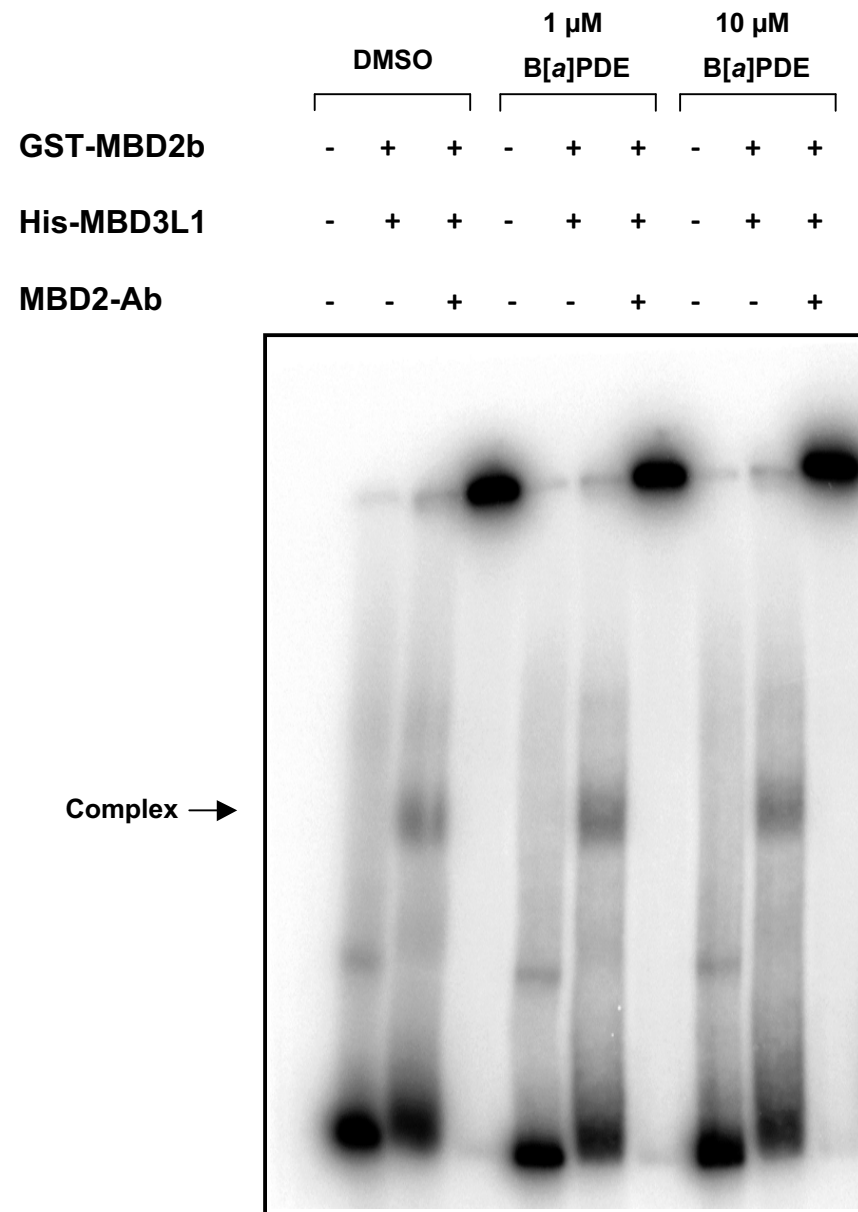

**Fig. S4**

Supplement: Figure S4 — Affinity of the MBD2b/MBD3L1 complex for methylated CpGs in the presence and absence of B[a]PDE-DNA adducts determined by gel mobility shift assay. A 55-mer oligonucleotide, containing 1-10 symmetrically methylated CpG dinucleotides, was treated with increasing concentrations of B[a]PDE, and subsequently subjected to electromobility gel shift assay, as described earlier (Rauch et al., 2006). Invariable formation of the MBD2b/MBD3L1 complex in the presence and absence of B[a]PDE-DNA adducts is indicated by an arrow. MBD2-Ab = Negative control, co-incubated with polyclonal antibody raised specifically against MBD2b protein. Representative result from the oligonucleotide with 10 methylated CpGs is shown. (0.23 MB PDF) [file pone.0010594.s004.pdf]

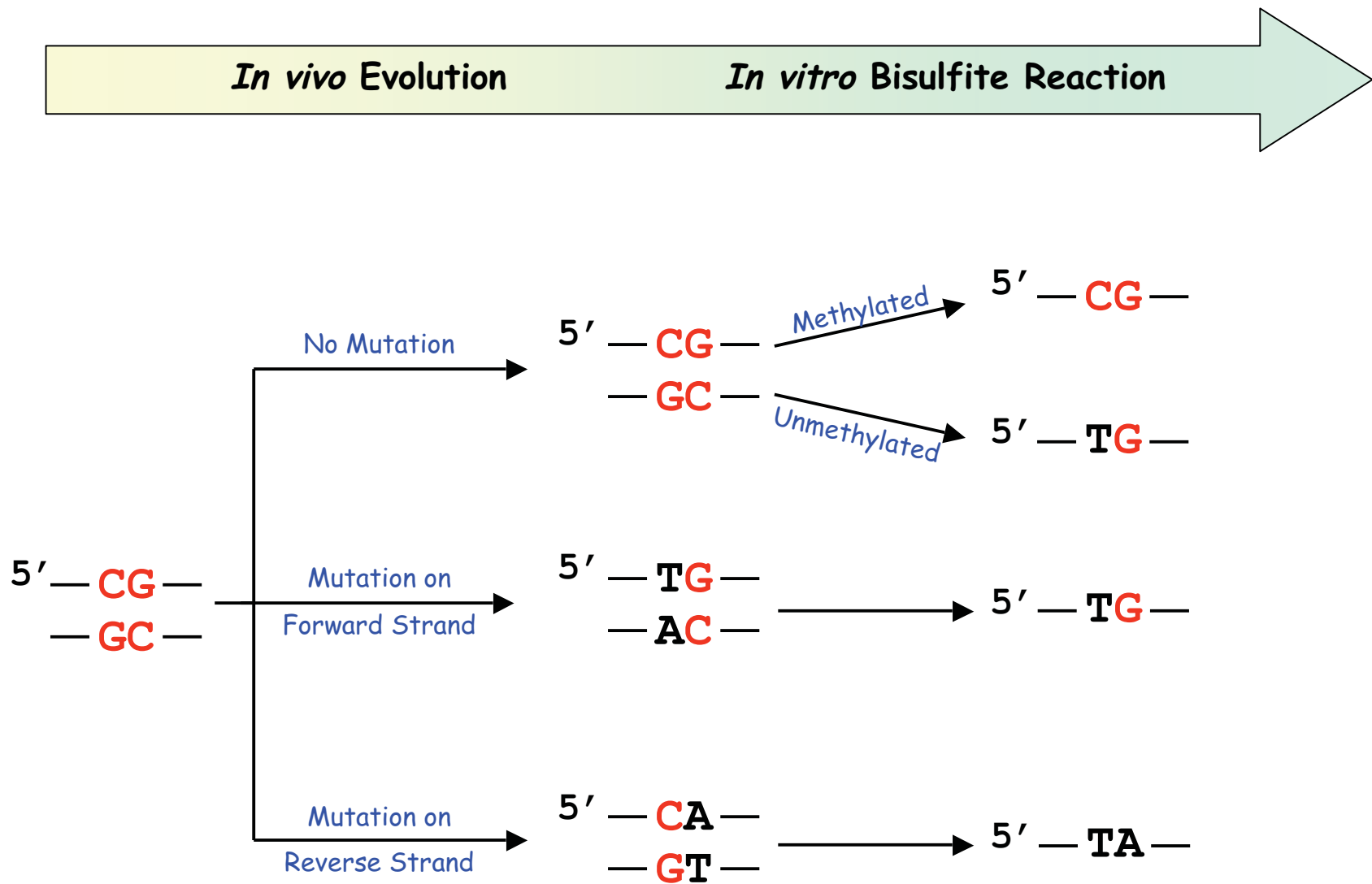

Fig. S5

Supplement: Figure S5 — Conceptual framework for the methylation detection assay in repetitive DNA elements. The assay is an adaptation of a published procedure (Yang et al., 2004), which involves primer amplification of the consensus sequences from the repetitive DNA elements followed by appropriate restriction digestion or direct sequencing (see, text for detailed information on methodology). Adopted from Ref. (Yang et al., 2004). (0.03 MB PDF) [file pone.0010594.s005.pdf]
